# The Role of Alcohol Use Disorders in the Development and Progression of Dementia

**DOI:** 10.1192/j.eurpsy.2024.1393

**Published:** 2024-08-27

**Authors:** A. H. I. Abu Shehab, T. Simona, A. B. Ciubară, D. C. Voinescu, C.-F. Buciu, A. Ciubară

**Affiliations:** ^1^Psychiatry Department, “Elisabeta Doamna” Psychiatric Hospital, Galati; ^2^Psychiatry Department, Carol Davila University of Medicine and Pharmacy, Bucharest; ^3^Orthopedics and traumatology Department; ^4^Rheumatology department, “Dunărea de Jos” University - Faculty of Medicine and Pharmacy, Galati; ^5^public health and management, University of Medicine, Pharmacy, Science and Technology of Târgu Mureș, Târgu Mureș; ^6^Psychiatry Department, “Dunărea de Jos” University - Faculty of Medicine and Pharmacy, Galati, Romania

## Abstract

**Introduction:**

In recent years, there has been an increase in interest and research into the link between alcohol use disorders (AUD) and dementia. Alcohol use disorders, which are characterised by excessive and problematic alcohol consumption, have been associated to a variety of detrimental health effects, including liver disease, cardiovascular difficulties, and cognitive impairments.

**Objectives:**

To explore the link between alcohol use disorders and dementia onset and progression, explaining probable causes and emphasising preventive approaches.

**Methods:**

The present study involved a thorough examination of relevant research papers, with a specific emphasis on longitudinal cohort studies, neuropathological observations, and biochemical interactions pertaining to the effects of alcohol on the brain. In addition to the aforementioned criteria, the review also took into account other complicating factors, including choices regarding lifestyle, genetic predisposition, and coexisting medical conditions.

**Results:**

The results indicate a strong association between prolonged and excessive alcohol consumption and a heightened susceptibility to the early onset of dementia. The mechanisms underlying alcohol-related neurological damage encompass direct neurotoxic effects of alcohol, thiamine shortage, and alcohol-related cerebrovascular illness. Moreover, it is worth noting that alcohol use disorder (AUD) has the potential to worsen the advancement of neurodegenerative processes in individuals already diagnosed with dementia.

**Conclusions:**

The association between AUD (Alcohol Use Disorder) and dementia is complex and involves multiple factors, presenting considerable difficulties in terms of clinical intervention and treatment. The use of early intervention strategies and public health initiatives focused on addressing alcohol use disorder (AUD) could have a significant impact on preventing or reducing the development of dementia.

**Disclosure of Interest:**

None Declared

